# Cytomegalovirus infection is associated with an increase in aortic stiffness in older men which may be mediated in part by CD4 memory T-cells

**DOI:** 10.7150/thno.58356

**Published:** 2021-03-31

**Authors:** Frances Kirkham, Alejandra Pera, Amanda M. Simanek, Aalia Bano, George Morrow, Bernhard Reus, Stefano Caserta, Helen E. Smith, Kevin A Davies, Chakravarthi Rajkumar, Florian Kern

**Affiliations:** 1 Department of Clinical and Experimental Medicine, Brighton and Sussex Medical School, Brighton, United Kingdom.; 2 Maimonides Institute for Biomedical Research of Cordoba (IMIBIC), Córdoba, Spain.; 3 Department of Cell Biology, Physiology and Immunology, University of Córdoba, Córdoba, Spain.; 4 Joseph J. Zilber School of Public Health, University of Wisconsin-Milwaukee, USA.; 5 Department of Informatics, School of Engineering and Informatics, University of Sussex, Brighton, United Kingdom.; 6 Department of Global Health and Infectious Diseases, Brighton and Sussex Medical School, Brighton, United Kingdom.; 7 Family Medicine and Primary Care, Lee Kong Chian School of Medicine, Nanyang Technological University Singapore.

**Keywords:** central aortic stiffness, pulse wave velocity, cardiovascular risk, human Cytomegalovirus (CMV), Memory T-cells

## Abstract

Human Cytomegalovirus (CMV) infection is associated with atherosclerosis, higher cardiovascular disease (CVD) risk, and an increase in memory T-cells (T_mem_). T-cells have also been implicated in CVD, independently of CMV infection. To better understand the CMV-associated CVD risk, we examined the association between CMV (IgG) serostatus and central aortic (carotid-to-femoral) pulse wave velocity (cfPWV), an early, independent predictor of CVD. We also investigated if such an association might be reflected by the distribution of T_mem_ and/or other T-cell subsets.

**Methods:** Healthy older volunteers (60-93 years) underwent routine clinical and laboratory evaluation, including assessment of cfPWV in eligible participants. Flow-cytometry was used to assess proportions of memory T-cells, CD28^null^ T-cells, and CMV-specific T-cells. The following associations were examined; CMV serostatus/cfPWV, CMV serostatus/proportion of T_mem_, proportion of T_mem_/cfPWV, CD28^null^ T-cells/cfPWV, and CMV-specific T-cells/cfPWV. Linear regression models were used to adjust for age, sex, socioeconomic status, smoking, waist-to-hip ratio, cholesterol, and blood pressure as required.

**Results:** Statistically significant positive associations were found (P-values for the fully adjusted models are given); CMV serostatus/cfPWV in men (P ≤ 0.01) but not in women, CMV serostatus/proportions of CD4 T_mem_ in men (P ≤ 0.05) but not in women; proportions of CD4 T_mem_/cfPWV among CMV seropositive (CMV+) people (P ≤ 0.05) but not CMV seronegative (CMV-) people.

**Conclusion:** CMV infection increases the CVD risk of older men by increasing cfPWV. This may be mediated in part by increased proportions of CD4 T_mem_, higher numbers of which are found in CMV+ older people and more so among men than women. Given the high prevalence of CMV worldwide, our findings point to a significant global health issue. Novel strategies to mitigate the increased CVD risk associated with CMV may be required.

## Introduction

A recent meta-analysis of 10 studies across a total of 34,564 participants concluded that human Cytomegalovirus (CMV) infection (i.e., the status of being anti-CMV IgG seropositive, or 'CMV+') increases the overall risk of cardiovascular disease (CVD) by 22% 1. However, the mechanisms underlying this association are not well understood. As CMV prevalence increases with age, the effects of CMV infection and aging are difficult to dissociate. CMV is often considered a confounder with respect to age-associated pathologies and vice versa. CMV infection has been directly associated with precursors to cardiovascular disease such as hypertension and atherosclerotic changes in a number of human and animal studies 2-4. For example, a mouse model of murine CMV (MCMV) infection demonstrated that, apart from causing an increase in blood pressure, MCMV was independently a co-factor for aortic intimal thickening 3. In humans, being CMV+ has also been found to be associated with increased blood pressure 4.

The exact mechanisms by which CMV infection contributes to CVD remain unclear, but there is increasing evidence that some of the major effects of CMV on the cardiovascular system are mediated by its impact on certain immune cell subsets, particularly antigen-experienced, memory-T-cells (T_mem_) 5-7. T-cells in general, and particularly CD4 T-cells, have been shown to be associated with atherosclerosis and atherothrombosis 8. Infection with CMV induces high frequencies of pro-inflammatory, CMV protein-specific effector T-cells 9, 10, but also increases the proportions of antigen-experienced CD4 and CD8 T-cells in general 9-11. Since vascular endothelial cells are a primary target tissue for CMV infection 12, CMV-specific T-cells may be targeted to vascular endothelium causing local damage; however, upregulated adhesion molecules on damaged endothelium may also attract non-CMV-specific T-cells 13, 14. A study in rats showed that intraperitoneal CMV infection, administered immediately after experimental vascular injury, significantly increased the neointimal response to that injury (i.e., intima to media ratio). Interestingly, this effect was independent of the presence of virus in the injured epithelium itself, suggesting that CMV-driven pro-inflammatory T-cells may non-specifically infiltrate injured vasculature 15.

Men and women differ with regards to age and CMV-associated changes to the immune system 6, 16, which may help explain sex differences in mortality from coronary heart disease and stroke 17. For example, in older men CMV infection induces larger proportions of highly differentiated CD4 and CD8 effector memory T-cells than in older women 6. Such effector cells may have a destabilizing effect on atherosclerotic plaques 8. Older women, by contrast, have more regulatory T-cells than older men, which have a stabilizing effect on atheromatous lesions 8. This might explain why atherosclerotic plaques are more stable in older women 18.

Compliance of the large elastic arteries is a well-established, early, and sensitive marker of cardiovascular risk, which can be measured non-invasively as central aortic pulse-wave velocity 19. It is calculated from the path length travelled by the pulse wave in the carotid-to-femoral region and its transit time, and referred to as carotid-to-femoral pulse-wave velocity (cfPWV). cfPWV has a well-documented positive correlation with cardiovascular outcomes such as heart attacks and stroke, independently of traditional risk factors 20, 21.

It was recently shown that CMV infection is associated with increased cfPWV in chronic kidney disease (CKD) 22, the latter affecting 11-13% of people worldwide 23. Since the prevalence of CMV infection (i.e. positive IgG serology) is about 80% in over 65 year-olds in European studies 24, 25 and even higher elsewhere 26, 27, any detrimental effect of CMV on cardiovascular health should be of major concern. Being able to demonstrate a similar effect of CMV infection on cfPWV in generally healthy older people would significantly corroborate the role of CMV as a driver of CVD and underscore its relevance to global health 20, 21. In the present work we examine if an association between CMV serostatus and cfPWV indeed exists in generally healthy older people. Also, in light of the suspected role of T-cells in the pathogenesis of atherosclerosis and cardiovascular disease 7, 8 and CMV's role in driving memory T-cell expansion 5, 6, we examine the distribution of different antigen-experienced T-cell subsets (T_mem_) as well as the size of the CMV-specific T-cell response across our population of older CMV+ and CMV- men and women. This allows us to examine the effect of T-cells on cfPWV that may be mediated by CMV serostatus and/or sex.

## Results

### In older men but not women, CMV-infection is associated with increased cfPWV

A possible association of CMV infection with an increase in cfPWV (as an indicator of CVD risk) was the primary focus of our study. Across our total cohort of older people (60-93 years) cfPWV (median) was within the reported normal range 28 with 8.9 m/s (IQR: 2.4 m/s) among 60-70 year-olds (N = 91), 9.8 m/s (IQR: 3.4 m/s) in 70-80 year-olds (N = 29), and 12.2 m/s (IQR: 3.3 ms) in 80-90 year-olds (N = 15). The prevalence of CMV infection was 54% among those assessed in the vascular lab (a study flow-chart is provided in Figure S1).

In CMV+ men the median cfPWV was 1.1 m/s higher than in CMV- men (P = 0.022). Among women, the difference was in the opposite direction but not statistically significant (Table 1 and Figure S2). According to published normal ranges, a difference of 1 m/s roughly corresponds to an age difference of about 5-10 years in the investigated age group 28. In order to confirm this important finding, we built linear regression models examining the association between CMV infection (positive IgG-serology) and cfPWV (Table 2) while adjusting for relevant covariates. In the entire cohort of N = 136 individuals (men and women) we observed no statistically significant association between CMV serostatus and cfPWV, neither in the crude nor adjusted models. However, since we had observed a significant difference in cfPWV between CMV+ and CMV- older men but not women, we added the interaction term 'CMV serostatus*sex' to the model. This term was significant at all levels of adjustment with P = 0.026 in the fully adjusted model, indicating that sex indeed influenced the effect of CMV-seropositivity. We subsequently stratified the model by sex. In the stratified but unadjusted model the mean cfPWV was 0.046 log10-units higher for CMV+ versus CMV- men (95% CI 0.001, 0.092; P = 0.046). In agreement with the observed median difference between these groups this translates into a difference of cfPWV of approximately 1.11 m/s (95% CI: 1.00 m/s to 1.24 m/s). The effect of CMV was slightly increased after adjusting for the demographic factors, age and socioeconomic status (SES) (P = 0.016), and even stronger after additionally adjusting for smoking pack-years and waist-to-hip ratio (WHR). After further adjustment for lying systolic blood pressure (SBP) and the ratio of total cholesterol over HDL-cholesterol (TC/HDL-C), cfPWV was 0.059 log10-units higher in CMV+ compared to CMV- men (95% CI: 0.024, 0.093; P = 0.001) translating into a difference of cfPWV of about 1.13 m/s (95% CI: 1.06 m/s to 1.24 m/s). In contrast to this, a negative but not statistically significant association was observed among women. Cohort characteristics relevant to the linear regression models are shown in Table 1. The complete, fully adjusted model is shown in the supplementary data (Table S1). There were no significant differences between CMV+ and CMV- individuals with regard to blood pressure control (Table 1 and Table S2) or blood pressure medications (Table S3) neither in the entire cohort nor among men or women separately. As would be expected in this case, adjusting for the use of blood pressure medications made no tangible difference to the fully adjusted regression models (Table S4).

It is well known that CMV infection skews T-cell subset distribution towards an advanced memory phenotype and it has been reported that this happens more so in older men than in older women 6, 16. Therefore, and because of the role of T-cells in cardiovascular disease it seemed pertinent to explore if the distribution of T-cell in terms of naïve and memory subsets in CMV+ men and women was reflective of the observed difference in cfPWV.

### The proportions of memory T-cell subsets differ between CMV+ and CMV- individuals in both men and women

Whole blood T-cell phenotyping data was available for a sub-cohort of N = 123 individuals who also had valid cfPWV measurements. Characteristics of this sub-cohort are reported in supplementary Table S5 (parameters used in linear regression models), Table S6 (additional information on blood pressure and smoking), and Table S7 (medications). In this sub-cohort, the association of CMV serostatus with cfPWV in men shown in Table 2 remained significant at all levels of adjustment. We used the surface markers C-C chemokine receptor 7 (CCR7) and CD45RA to identify naïve T-cells (CCR7+CD45RA+, T_NA_), as well as central memory (CCR7+CD45RA-, T_CM_), effector memory (CCR7-CD45RA-, T_EM_), and revertant memory T-cell subsets (CCR7-CD45RA+, T_EMRA_), collectively referred to as T_mem_ (Figure S3) 29.

**Figure 1:**
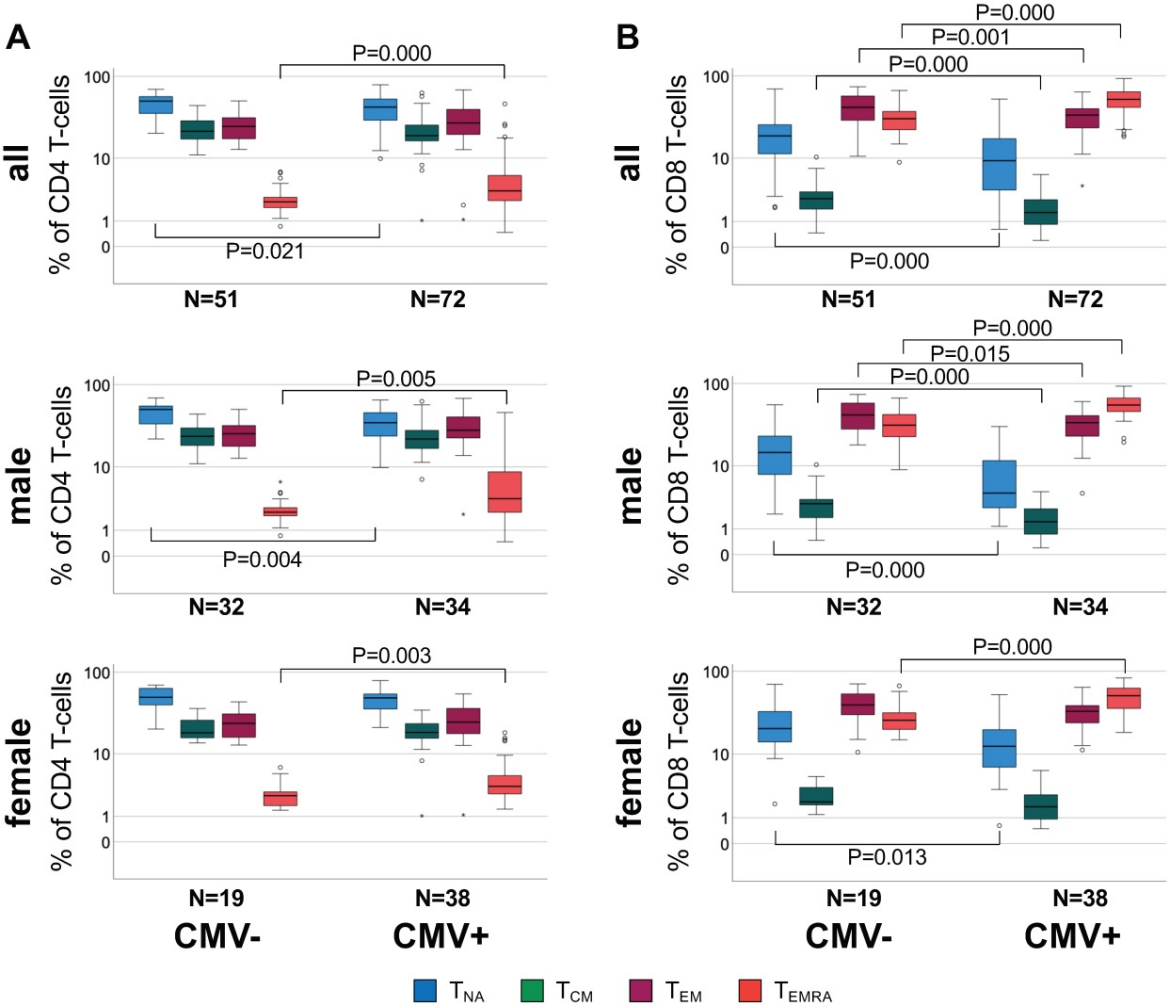
** Differential distribution of canonical memory T-cell subsets in different sub-groups defined by CMV serostatus and sex.** A. Box plots show the distribution of CD4 T_NA_, T_CM_, T_EM_, and T_EMRA_ subsets in CMV- and CMV+ individuals among the entire cohort (top), men (middle) and women (bottom). B. Box plots show the distribution of the corresponding CD8 T cell subsets in each group. Box plots show median and interquartile range, outlier limits (whiskers, LQ-1.5*IQR, UQ+1.5*IQR), outliers (o) and extreme values (_*_). Significant differences are indicated by P-values.

We examined CD4 and CD8 T-cell subset differences (i.e., relative population size) between CMV+ and CMV- individuals in the entire cohort but also separately among male and female participants, because CMV serostatus was associated with a difference in cfPWV in men but not women (Figure 1).

When comparing CMV+ to CMV- individuals, significant subset differences were found with regards to CD4 T_NA_ and CD4 T_EMRA_ (Figure 1A, top) and all CD8 subsets (Figure 1B, top). Upon stratification by sex these differences seemed to be stronger among men than women (relative difference of median subset size, only significant differences are listed). In CMV+ compared to CMV- men, CD4 T_NA_ were 30.5% lower (P = 0.004), and CD4 T_EMRA_ 63.3% higher (P = 0.005) (Figure 1A, middle), CD8 T_NA_, T_CM_, and T_EM_ were 71.1% (P = 0.000), 51.2% (P = 0.000), and 19.4% (P = 0.015) lower, respectively, however, CD8 T_EMRA_ cells 77.8% higher (P = 0.000) (Figure 1B, middle). In CMV+ compared to CMV- women, CD4 T_EMRA_ cells were 40.1% higher (P = 0.003) (Figure 1A, bottom), CD8 T_NA_ were 39.5% lower (P = 0.013) and CD8 T_EMRA_ cells 96.6% higher (P = 0.000) (Figure 1B, bottom).

Statistically significant differences between men and women regarding T_mem_ subset distribution were observed in the entire cohort and among CMV+ individuals but not CMV- individuals (Figure S4). In CMV+ men CD4 T_NA_ were 28.4% lower (P = 0.002) and CD8 T_NA_ 66.1% (P = 0.001) lower than in CMV+ women (relative difference of median subset size).

**Figure 2:**
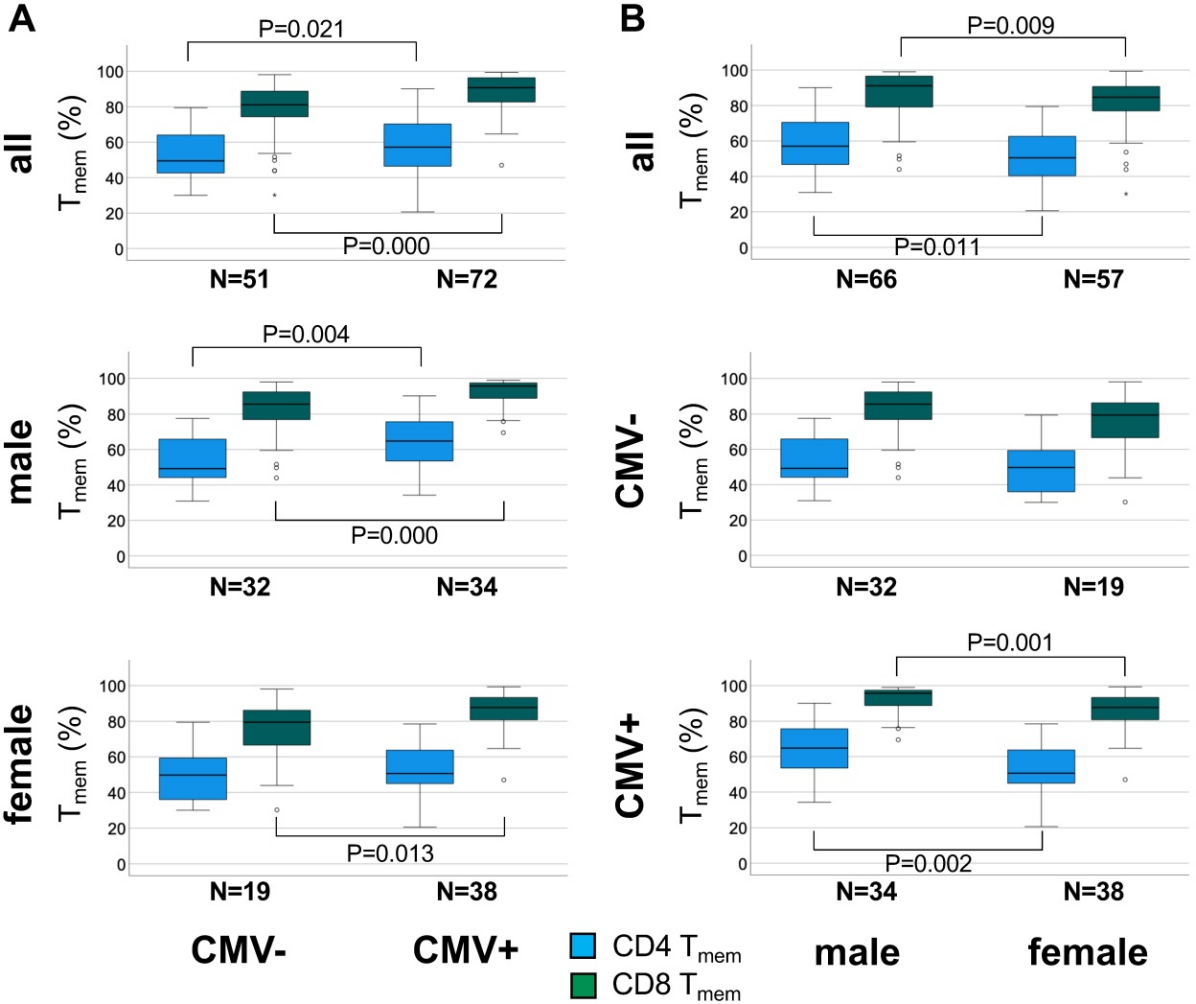
** The percentage of antigen-experienced CD4 T_mem_ cells is higher in CMV+ than CMV- individuals among men but not women.** A. Box plots show the proportions of CD4 and CD8 T_mem_ in the entire cohort (top), men (middle), and women (bottom). B. Box plots show the proportions of CD4 and CD8 T_mem_ in the entire cohort (top), CMV- individuals (middle), and CMV+ individuals (bottom). Box plots show median and interquartile range, outlier limits (whiskers, LQ-1.5*IQR, UQ+1.5*IQR), outliers (o) and extreme values (_*_). Significant differences are indicated by P-values.

The overall effect of CMV infection on the canonical memory T-cell subsets appeared to be well captured by either the size of the naïve subset (T_NA_) or the T_mem_ subset (T_CM_, T_EM_, and T_EMRA_). Since T_NA_ and T_mem_ subset sizes are reciprocal, only the size of the T_mem_ subset was used for further analysis. Figure 2 summarizes the differences in CD4 and CD8 T_mem_ subset size between CMV- and CMV+ individuals in the whole cohort, among men and women.

In the entire sub-cohort CD4 T_mem_ were 15.7 % higher (P = 0.021) in CMV+ compared to CMV- individuals (relative median differences given) (Figure 2A, top). Among men the corresponding difference was 31.6% (P = 0.004) (Figure 2A middle), but among women only 1.9% (n.s.) (Figure 2A bottom), indicating a much stronger effect of CMV in men than in women. In the entire cohort CD8 T_mem_ were 12.2% higher (P = 0.000) in CMV+ compared to CMV- individuals (Figure 2A, top) but the corresponding difference among men and women was very similar (12.0%, P = 0.000 and 10.4%, P = 0.013, respectively) (Figure 2A, middle and bottom).

When directly comparing the sexes among CMV+ people, the CD4 T_mem_ subset was 27.9% bigger (relative median difference) in men than in women (P = 0.002, Figure 2B, bottom), however, the CD8 T_mem_ population was only 9% larger in men than in women (P = 0.001).

We wondered therefore if the significant difference in CD4 T_mem_ between CMV+ and CMV- men (which was not found in women) might explain the association between CMV serostatus and pulse wave velocity that we had observed in men (but not in women). In order to be sure that the difference in CD4 T_mem_ was truly associated with CMV serostatus and not, for example age, we first tested the effect of potential confounders in a linear regression model (Table 3). The model was initially run on the entire sub-cohort and was adjusted for age, sex, SES, smoking, and WHR, but not for SBP and cholesterol, as these last two covariates have no known relationship with T_mem_ subset distribution. To account for the observed sex differences revealed by our memory T-cell subset analysis, the model was subsequently stratified by sex, which confirmed a significant effect of CMV on CD4 T_mem_ in men but not women We also tested the effect of CMV serostatus on CD8 T_mem_, which, as expected, was similar in both sexes and significant at all levels of adjustment (Table S8).

### Antigen-experienced CD4 T-cells correlate positively with cfPWV in CMV+ but correlate negatively in CMV- older people

**Figure 3:**
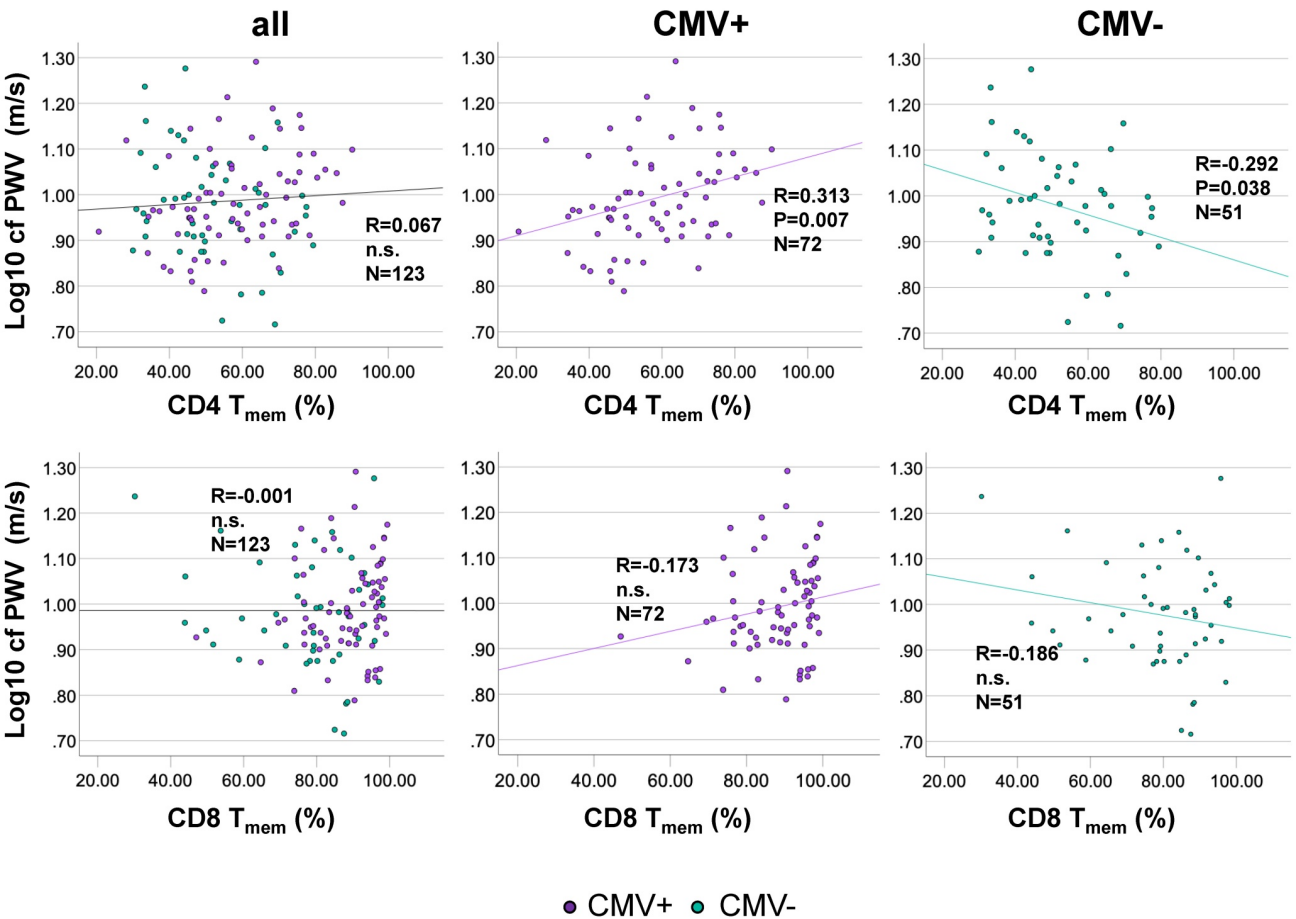
** The percentage of antigen-experienced CD4 T_mem_-cells correlates positively with cfPWV in CMV+ but correlates negatively in CMV- people.** Scatterplots show the relationships between the percentage of antigen-experienced CD4 T_mem_-cells (A), or CD8 T-cells (B) and cfPWV. The entire cohort is shown on the left, CMV+ individuals in the middle, and CMV- individuals on the right. cfPWV was Log10-transformed to improve visualization. Regression lines denote the results of regression on T_mem_. The Pearson correlation coefficient (R) is shown with each regression line.

We next assessed whether the proportions of CD4 T_mem_ were associated with cfPWV. Bivariate correlation analysis indicated that among CMV+ individuals CD4 T_mem_ population size was moderately positively associated with cfPWV, but not in the whole cohort. Among CMV- individuals CD4 T_mem_ population size was moderately negatively associated with cfPWV (Figure 3A). No significant correlation between the proportions of CD8 T_mem_ and cfPWV was identified in the entire cohort or after stratification by CMV-status (Figure 3B). As expected in this cohort of over 60 year-olds, no significant changes in CD4 T_mem_ or CD8 T_mem_ were observed as a function of age (not shown).

The association between CD4 T_mem_ and cfPWV was confirmed in a linear regression model (Table 4). The model was run in the entire sub-cohort as well as after stratification by CMV status based on the differences observed between CMV+ and CMV- people. Adjustment for covariates in this model was analogous to the first model testing the effect of CMV on cfPWV. As expected, in the whole cohort, no significant effect of CD4 T_mem_ on cfPWV was identified, neither crude nor after adjustment for CMV or at any further level of adjustment (models 2-5). Splitting the model by CMV serostatus (in analogy to Figure 3) identified a statistically significant positive association between CD4 T_mem_ and cfPWV among CMV+ people but a significant negative association among CMV- people. However, following adjustment, the positive association among those who were CMV+ remained significant but the beta regression coefficient was somewhat reduced from 0.249 (unadjusted) to 0.181 (fully adjusted). Since both cfPWV and T_mem_ were log transformed for inclusion in the model, the beta coefficients in Table 4 are given in log units as well. A beta of 0.181 for CMV+ people (fully adjusted model) translates into an approximate increase of 0.3 m/s of cfPWV for every 10% increase of CD4 T_mem_. This clearly is a sizeable effect (compare Figure 3, which corresponds to the unadjusted model). The negative association between CD4 T_mem_ and cfPWV among CMV- individuals lost significance following adjustment. In order to avoid collinearity, these models were not adjusted for sex, since the effect of sex is at least partially accounted for by the difference in size of the CD4 and CD8 T_mem_ populations between men and women.

### CD28^null^ T-cell subset size is not significantly associated with cfPWV

**Figure 4:**
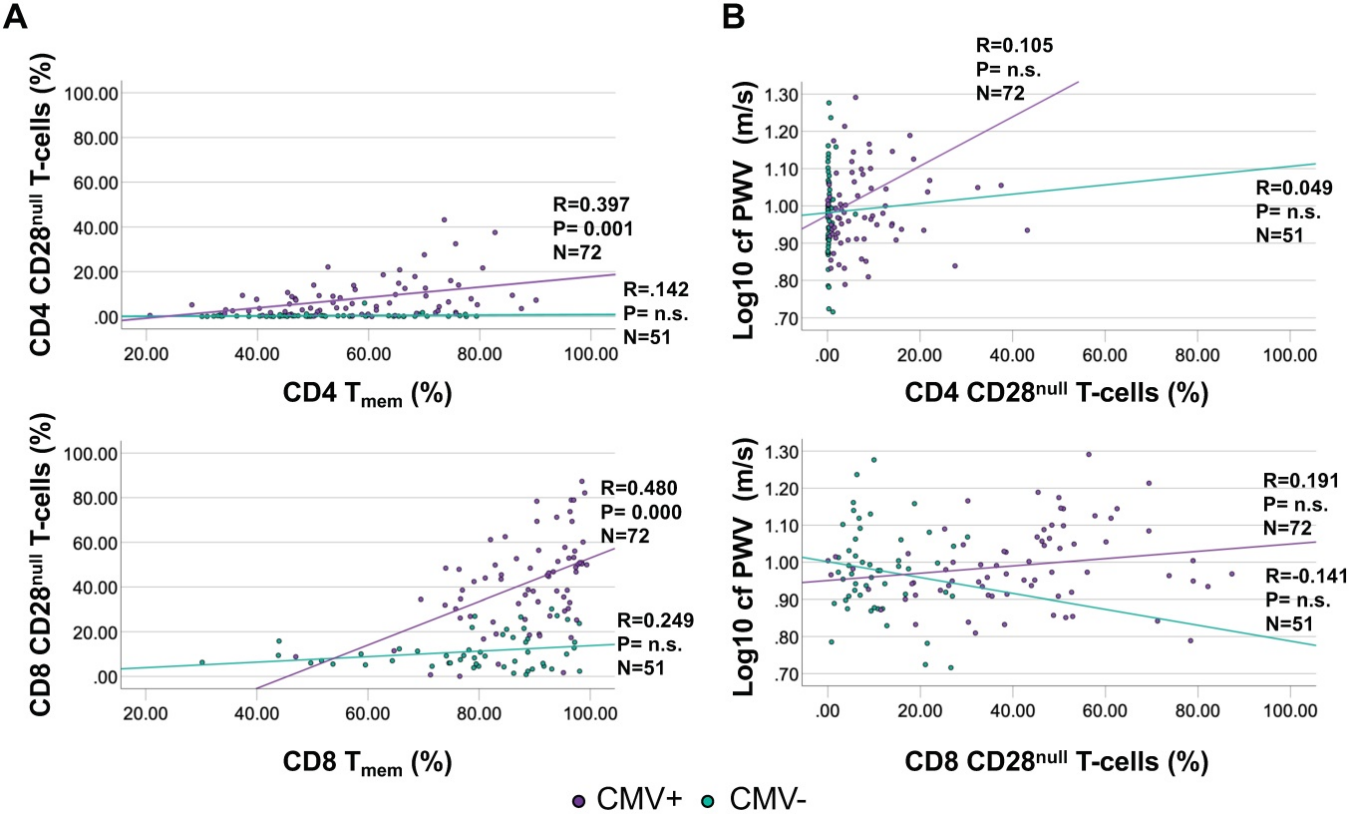
** The proportions of CD4 and CD8 CD28^null^ T-cells show a moderate correlation with T_mem_ among CMV+ people but do not correlate with cfPWV.** CD28^null^ T-cells were measured by flow-cytometry (gated as CD28^null^/CD27- T-cells). A. Correlations between CD28^null^ CD4 (top) and CD8 (bottom) T-cells and T_mem_ are shown separately for CMV+ and CMV- people. B. Correlations between CD28^null^ CD4 (top) and CD8 (bottom) T-cells and cfPWV. Regression lines denote the results of regression on T_mem_ or CD28^null^ T-cells. The Pearson correlation coefficient (R) is shown with each regression line.

As a T-cell subset of particular interest, CD28^null^ T-cells are implicated in vascular damage and particularly in destabilizing atherosclerotic plaques 30. CD4 CD28^null^ T-cells are strongly associated with CMV infection, with many actually being CMV-specific and occurring only in small frequencies in CMV- individuals 31. CD28^null^ T-cells were gated as CD3+/CD8+ or CD4+/CD28^null^/CD27- 31. While no significant correlation was observed between CD28^null^ T-cells and T_mem_ among CMV- individuals (CD28^null^ CD4 T-cell proportions were very low among CMV- people), the correlation was moderate among CMV+ individuals (Figure 4A). However, there was no significant correlation between CD4 or CD8 CD28^null^ T-cells and cfPWV, neither among CMV- nor among CMV+ individuals (Figure 4B). Because no meaningful association between CD28^null^ T-cells and cfPWV was suggested by the data, their role in causing increased cfPWV was not explored further.

### CMV-specific T-cells are not directly associated with cfPWV

We also examined if the number of CMV- specific CD4 or CD8 T-cells were associated with cfPWV (the analysis being obviously limited to CMV+ individuals). These cells are known to predominantly have an advanced memory phenotype 32. We measured activated CMV-specific CD4 and CD8 T-cells following stimulation of fresh PBMC with CMV protein-spanning peptide pools 33, 34. The proteins for stimulation were selected from a panel of 19 proteins that had been found to be representative of all CMV T-cell target antigens in a mixed ethnicity population 35. However, 3 proteins against which we had not detected a single response in White British people in a recent study were left out 9, 10. The remaining 16 proteins were arranged in 14 stimulation pools (Table 5). Intracellular Interleukin-2 (IL-2), Tumor-necrosis factor (TNF), and Interferon-gamma (IFN-γ) were used separately or in combination to identify activated cells and the sum of the responses to all pools was used as a compound measure of T-cell responsiveness 35. This avoids the significant bias introduced by measuring responses to single proteins 36. Complete measurements were available for 69 CMV+ individuals who also had valid cfPWV measurements (33 male and 36 female). Figure S5 shows the distribution of CD4 and CD8 T-cell responsiveness (i.e., presence or absence of a response) across the tested peptide pools in our study.

**Figure 5:**
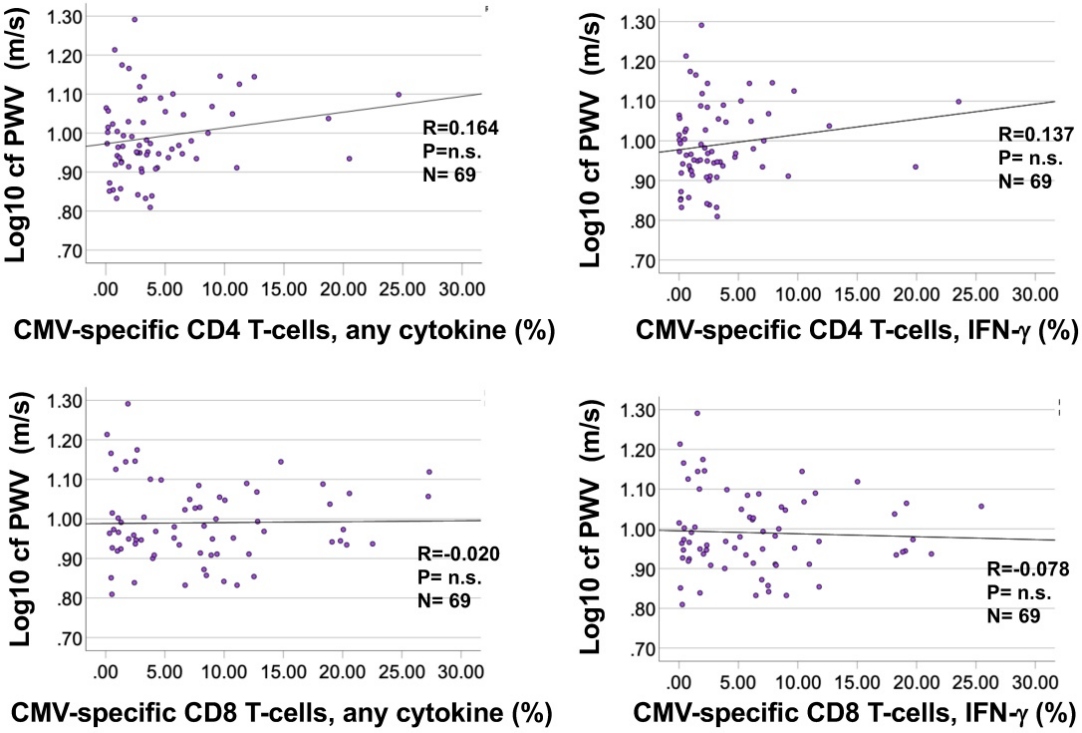
** The frequencies of CMV-specific CD4 and CD8 T-cells do not correlate with cfPWV.** Responses were measured by intracellular cytokine staining following stimulation of fresh PBMC with 16 CMV protein-spanning peptide pools arranged in 14 stimulation pools. For each protein the percentage of cells expressing each activation marker (IL-2, TNF, or IFN-γ) or at least one of them was determined. The summated responses across all 14 CMV-peptide stimulation pools are plotted against cfPWV. Response size was determined based on the presence of at least one activation marker (A) or (the usually dominant) IFN-γ only (B).

Bivariate correlation analysis did not identify significant associations between the percentages of CMV-specific CD4 or CD8 T-cells and cfPWV (Figure 5A and B) for any of the individual stimulation pools (not shown). No further analyses were carried out to test associations between CMV-specific T-cells and cfPWV.

## Materials and methods

### Ethics Statement

The study was approved by the UK National Research Ethics Service (NRES) 'London Centre' (Reference 13/LO/1270). Written informed consent was obtained from all participants. The study was conducted in accordance with the Declaration of Helsinki.

### Participants and samples

Participants were recruited through general practices (GP) in Southern England with help of the primary care research network (PCRN). Inclusion criteria were; White British ethnicity and age 60 years or older; exclusion criteria were; known immunodeficiency (including HIV infection), organ transplantation, use of immunosuppressive or immune-modulating drugs within the last year (excluding acetylsalicylic acid ≤ 100 mg/day), cancer or treatment for cancer within the previous 5 years, insulin dependent diabetes, moderate or advanced renal failure, liver disease, endocrine disorders (except corrected thyroid dysfunction), manifest autoimmune disease, dementia/mental incompetence, known alcoholism or other drug abuse, acute infection or illness in the last 4 weeks, raised body temperature (> 37.5 °C), moderate or severe heart failure (NYHA III or IV), inability to lie flat. Individuals with typical, age-related cardiovascular morbidity were not excluded. A flow-chart outlining recruitment and response data is shown in Figure S1. Further details are available in the online supplement.

### Participant data and sample collection

Information collected at the main appointment included demographic and lifestyle factors including a detailed smoking history and socioeconomic status (SES), prior medical history, and medications. All participants also underwent a physical examination. Additional measurements included height, weight, waist and hip circumference, body composition, blood pressure, and vascular stiffness. Blood samples were taken by peripheral venepuncture.

### CMV Serology

CMV immunoglobulin G (IgG) serology (Architect CMV IgG, Abbot, Maidenhead, UK) was performed at the Brighton and Sussex University Hospital Trust (BSUHT) virology laboratory using the same routine assay as used for all hospital patients. Individuals whose serum samples exceeded the assay threshold for positive IgG are referred to as 'CMV seropositive' or 'CMV-infected'.

### Medications

A detailed medical history included all current medications. An overview of common medications is provided for the entire cohort (Table S3) and separately for the sub-cohort with T-cell phenotyping data (Table S7).

### Smoking

A detailed smoking history was collected which accounted for all types of tobacco smoking. Smoking other than cigarettes was translated into cigarette equivalents as follows; large cigars (4), small cigars/cigarillos (2), self-rolled cigarettes (1), pipes per bowl (1.5) 37. The smoking history was represented by (cigarette) pack-years. Separate periods of smoking interrupted by periods of non-smoking were summed to determine the total number of years that an individual had smoked in their lifetime. Additional information on smoking is provided in Table 1 and Table S2 for the entire cohort as well as Table S5 and Table S6 for the sub-cohort with phenotyping data.

### SES

Participants were asked to report their last job title and a description. We used the UK National Statistics Socio-economic Classification 2010 38 to generate job title codes. The ONS NS-SEC online coding tool 39 was then used to assign 'analytic categories' and 'operational categories' for each participant based on the job title code. This tool utilises the job title code and takes into account whether a person is self-employed or unemployed and if they have any supervisory roles. Ambiguous cases were assigned the highest of any possible codes. We used the operational category to represent SES in our analysis.

### PWV

Assessment of carotid-to-femoral PWV (measured in m/s) was carried out using the Complior® system (Alam Medical, Paris, France) at room temperature with the patient supine and after 10 minutes of resting. Patients had been asked to refrain from smoking, eating or drinking caffeinated drinks for 3 hours and drinking alcohol for 10 hours before measurements. Please find additional details in the online supplement.

### Resting blood pressure

Resting blood pressure was measured in duplicate using standardised BP monitors (OMRON705-IT, Omron Electronics Ltd., Milton-Keynes, UK).

### Laboratory evaluation of blood samples

Participant samples were treated like routine clinical samples and sent to the BSUHT pathology laboratory immediately after venepuncture. Analysis included routine full blood counts and fasting lipid profiles (total cholesterol, HDL cholesterol, triglyceride, TC/HDL ratio, non-HDL cholesterol).

### Whole blood T-cell phenotyping

Fresh whole blood was stained with fluorescence-labelled monoclonal antibodies: CD45, CD3, CD4, CD8, CCR7, CD45RA, in a routine 'lyse and wash' protocol (for details see online supplement).

### CMV peptides

Overlapping peptide pools representing 16 CMV different proteins arranged in 14 pools were used in T-cell stimulation assays (for details see online supplement).

### CMV reactivity of T-cells

Freshly obtained PBMCs from CMV+ individuals were stimulated overnight (16 h) with overlapping CMV protein spanning peptide pools and subsequently stained and acquired by flow-cytometry. Surface markers included CD3, CD4, CD8, CD45RA, CCR7. Intracellular markers included IL-2, TNF, and IFN-γ (activation markers). The gating strategy is shown in Figure S6. Based on the positive control stimulation (SEB) a 'response region' was defined in a given plot (e.g., CD4 versus IFN-γ) in which cytokine positive events (IFN-γ in the example) were located. A positive cytokine response following stimulation with peptide pools was then identified in an analogous plot as a clustered population of activated cells displaying the activation marker in question (i.e. IFN-γ in the example) provided that (i) the clustered population of activated events occurred in the response region, and (ii) the cluster of activated cells comprised at least 0.01% of the reference population (CD4 or CD8 T-cells) after subtracting the corresponding percentage of cytokine positive events found in the same region in an unstimulated sample (negative control).

### Statistical analysis

Statistical analysis was carried out using the SPSS 25 software package (IBM, London, UK). Our entire analytic sample (cohort) of N = 136 participants contained only individuals with valid cfPWV measurements who also had complete medical histories/data for all parameters of interest (hip and waist circumference, pack-year smoking history; socioeconomic status, pulse pressure, total and HDL cholesterol). Whole blood T-cell phenotyping was further available for a sub-cohort of N = 123 individuals. Non-parametric tests (Mann-Whitney U Test) were used to compare cfPWV and T-cell subset sizes between groups of individuals. Linear regression models were built to examine the association between CMV serostatus and cfPWV. For use in such models a number of parameters were log-transformed to improve normality, e.g., cfPWV and systolic blood pressure. Additional details of the statistical procedures are provided in the online supplement.

## Discussion

Our main aim was to investigate whether CMV infection (i.e., positive IgG serostatus) is associated with increased central aortic stiffness in generally healthy people over 60 years of age. Importantly, we identified a positive association of CMV serostatus with increased cfPWV among men indicating an increase in CVD risk 19, 28. Because of the known associations of (i) CMV infection with T_mem_ expansions 32 and (ii) T_mem_ subsets with atherosclerosis 7, 8 we examined if T_mem_ subset size and distribution in our cohort differed between CMV+ and CMV- individuals among men and women and might be associated with the difference in cfPWV that we had observed between CMV+ and CMV- men. Indeed, significant differences in CD4 T_mem_ distribution between CMV+ and CMV- individuals were only found among men, which raised the question of whether CD4 T_mem_ cells are associated with increased cfPWV and so could help explain the differential effect of CMV on cfPWV in men and women.

Whereas in the whole cohort no correlation between CD4 or CD8 T_mem_ subset size and cfPWV was identified, a positive correlation between CD4 T_mem_ and cfPWV was seen among CMV+ people but a negative correlation between these parameters was found in CMV- people. Linear regression confirmed a significant positive association between CD4 T_mem_ population size and cfPWV in CMV+ individuals at all levels of adjustment, however, the negative association between the same parameters among CMV- individuals was lost. Meanwhile, regarding CD8 T_mem_ population size there was still no significant association with cfPWV after stratifying by CMV serostatus.

Prompted by published work 40, 41 and the known, close link between CMV infection and the proportions of CD28^null^ T-cells 30, 31 we also examined correlations between CD28^null^ T-cell population size and cfPWV. We were, however, unable to confirm a significant effect of CD28^null^ CD4 or CD8 T-cells on cfPWV. Since a CMV-associated effect of T-cells on cfPWV could also be mediated by CMV-specific T-cells, we additionally tested associations between CMV-specific CD4 or CD8 T-cells and cfPWV but no significant association was elucidated either.

Our results therefore show that (i) CMV infection is associated with statistically significant increases in cfPWV and CD4 T_mem_ in older men but not women, (ii) that CMV is linked to larger increases in CD4 T_mem_ in men than in women, and (iii) that the proportions of CD4 T_mem_ have a moderate but statistically significant positive association with cfPWV among CMV+ individuals. This raises the possibility that the effect of CMV infection on cfPWV is at least partly mediated by CD4 T_mem_ and observable in men but not women because upon CMV infection CD4 T_mem_ do not increase to the same extent in women as in men.

To our knowledge, sex as a possible modifier of the association of CMV with increased CVD risk has never been reported nor specifically investigated. For example, a very recent analysis of five longitudinal older cohorts concluded that CMV infection is not associated with all-cause or cardiovascular mortality in community-dwelling older adults 42. However, authors did not consider the possibility of differential effects of CMV in men and women, different proportions of CMV+ individuals among men and women, or differences in cardiovascular disease burden in those dying from other causes. Had we not considered differential effects of CMV in men and women in our study, we would not have detected an effect of CMV on cfPWV as it seemed to work in opposite directions in the two sexes. Interestingly, the BELFRAIL study of over 80 year-olds in Belgium 43 suggested that being CMV+ might have a protective effect in older women with regards to all-cause mortality at 3.3 years. The mechanism behind this effect has so far remained unclear but its presence appears to confirm an important difference in how male and female immune systems handle this virus. Identifying and understanding sex-specific pathology therefore is extremely important in order to allow proper tailoring of preventative and therapeutic strategies according to sex. There are many examples of sex differences with regards to disease susceptibility and outcomes in particular in the field of autoimmunity and inflammation 44. Immune system differences between the biological sexes are plentiful and may be mediated by sex hormones, however, often persist beyond the menopause 45-47. Data from mouse models have confirmed sex differences in the way CMV affects the immune system by showing that male mice produce more inflammation in response to murine CMV (MCMV) infection than female mice. This seems to be related to higher expression of Toll-Like-Receptor 9 (TLR9) in males 48. Higher expression of TLR9 results in a stronger innate and adaptive immune response against MCMV, which drives advanced T-cell differentiation. If the same is true in humans, men will produce more inflammation in response to CMV infection than women, contributing to increased numbers of memory T-cells and increased vascular damage in later life.

With regard to vascular damage in the context of CMV infection it is important to consider that EC are a primary target tissue of CMV infection. CMV-specific CD4 memory T-cells can damage and infiltrate infected endothelium because they display a highly differentiated memory phenotype with increased secretion of pro-inflammatory cytokines 13, 14. Changes induced by endothelial inflammation include the upregulation of adhesion molecules, which, importantly, will also allow non-CMV-specific CD4 T-cells to infiltrate these areas, since adhesion molecules do not discriminate between T-cells of different antigen-specificity. In that sense, CMV-infection/CMV-specific T-cells may act as enablers allowing large numbers of activated memory/effector T-cells with a wide spectrum of antigen-specificities to infiltrate the vasculature 49. By virtue of unspecific by-stander activation these might contribute to vascular inflammation, sclerosis and, ultimately reduced elasticity. This could explain the observed positive association of CD4 T_mem_ with increased cfPWV in CMV+ people observed in our study.

The CD28^null^ subset of T_mem_ has attracted particular interest in the field of atherosclerosis, in particular coronary artery disease 30, 31. However, we were unable to identify an association between CD28^null^ T-cells and cfPWV despite the fact that an association of CD4 CD28^null^ T-cells with cfPWV was previously reported in patients with ANCA-associated vasculitis (AAV) 41. It is possible that the presence of autoimmune disease is an important amplifier of this association 50.

Regarding the role of CMV-specific T-cells, the CMV-specific portion of the entire T-cell repertoire as such is very difficult to quantify. It would essentially require testing responses against all potential CMV T-cell targets 35. Using the summed response to a range of peptide pools representing the most frequently recognized CMV-proteins (as in the present study) is far better than focusing on single or just a few proteins 35, 36, however, still does not mean that CMV-specific T-cell immunity was captured in a representative way. This could be one reason why the size of the CMV-specific T-cell response was not associated with cfPWV in our study. However, given that not even a weak association was found a more plausible explanation might be that, as discussed above, CMV infection and CMV-specific T-cells only initiate the process of vascular infiltration by other effector/memory T-cells but that CMV-specific T-cells do not themselves cause most of the damage. Interestingly, others have reported a positive correlation between the proportions of CD8 T-cells recognizing the CMV UL83 protein and PWV in a cohort of N = 123 CMV+ Korean individuals (including patients with hypertension, coronary artery disease, diabetes mellitus, and healthy subjects), however, this association was much weaker still than the one we found between CD4 T_mem_ and cfPWV 40. Based on the available data it would appear therefore that the direct contribution of CMV-specific T-cells to vascular stiffness is small.

Our study may be limited somewhat by a low participant response rate to the invitation which could affect the generalizability of our results to a degree. The reasons for this low rate of response potentially included the need to be fasting on arrival, the advised duration of the appointment of several hours, and the necessary travel to the investigation unit. These requirements might have biased the study in such a way that fitter individuals were more likely to self-select for participation. The fact that (i) we instructed GP practices to recruit generally healthy individuals and (ii) CMV status was unknown largely avoided any referral bias with respect to the presence of CMV-associated pathology or vascular changes. However, since in older people CMV infection has been associated with frailty 51 and chronic conditions for which individuals were excluded (such as insulin-dependent diabetes) 52, CMV+ individuals, in particular, might have been less likely to participate. However, such an effect would probably have biased the results against the detected association because frailty is itself associated with higher aortic pulse wave velocity 53 and so is diabetes at older ages 54. The percentage of CMV seropositive individuals in the study (54%) was marginally below but very close to the published CMV prevalence in the UK among 40-79 year-olds (59%) 55 and cfPWV measured in our study matched published normal values very closely 28. The slightly lower than expected seroprevalence was in line with our previous study in healthy older people in the same region of the UK 10 and, given that CMV infection is associated with a range of morbidities, especially at older ages 4, 51, 52, was probably the result of selecting older participants that were generally fit and well.

It was part of the study design to include only White British people in order to avoid differences in genetic background between CMV+ and CMV- participants affecting the results. This is a limitation with regards to generalizing our results to other ethnic groups and future confirmatory studies should include people of diverse biogeographical ancestry, however, these will have to be by orders of magnitude larger.

## Conclusions

In conclusion, an association of CMV infection with increased central aortic PWV in generally healthy, White British older males is an alarming finding. If this association extends to healthy older men in general it would represent a significant global health issue, given that CMV prevalence is as high or higher than 90% in large parts of the world 26. In order to address this problem, specific preventative strategies may be needed to mitigate the additional CVD risk of CMV+ males. This might involve strategies to prevent CMV-driven CD4 T_mem_ expansion, for example. It was previously shown in mice that treatment with Valaciclovir can reverse MCMV-specific CD8 T-cell expansion 56. Whether a similar approach would work for CD4 T-cells and in humans has yet to be established. Another potentially interesting agent could be Rapamycin, a safe and widely used immuno-modulatory drug that was recently shown to have anti-aging properties, enhance immune function and reduce infections and inflammation in older people 57, 58 but also to possess anti-CMV activity 59. In order to be able to develop the best therapeutic strategies in future, it is paramount that awareness of the health risks associated with chronic CMV infection increases among scientists and physicians. Further studies should be conducted to confirm our findings and identify the most suitable therapeutic targets in this complex interplay of CMV infection and the (aging) immune system.

## Supplementary Material

Supplementary figures and tables.

## Figures and Tables

**Table 1:** Characteristics used in linear regression models (entire study cohort)

	All			Male		Female			
	CMV- (N = 60)	CMV+ (N = 76)	P	CMV- (N = 36)	CMV+ (N = 36)	P	CMV- (N = 24)	CMV+ (N = 40)	P
cfPWV (m/s), median (IQR)	9.2 (3.2)	9.4 (2.9)	n.s.	9.0 (2.4)	10.1 (2.5)	0.022	9.7 (4.8)	8.9 (2.9)	n.s.
Age years, mean ± STD	69.2 ± 7.4	70.2 ± 7.1	n.s.	69.6 ± 7.6	70.7 ± 8.5	n.s.	68.5 ± 7.2	69.7 ± 5.5	n.s.
SES, median (IQR)	4.3 (3.0)	5.5 (4.8)	n.s.	4.1 (3.9)	6.0 (5.7)	n.s.	5.5 (4.5)	4.7 (3.1)	n.s.
Smoking (pack-years), median (IQR)	0.5 (10.1)	0.2 (7.0)	n.s.	0.3 (12.5)	2.4 (22.0)	n.s.	0.6 (9.4)	0.0 (3.2)	n.s.
WHR, mean ± STD	1.0 ± 0.2	0.9 ± 0.1	n.s.	1.0 ± 0.2	1.0 ± 0.1	n.s.	0.9 ± 0.1	0.9 ± 0.1	n.s.
TC/HDL-C, mean ± STD	3.3 ± 1.0	3.2 ± 0.8	n.s.	3.6 ± 1.0	3.3 ± 0.8	n.s.	3.0 ± 0.9	3.1 ± 0.8	n.s.
SBP lying (mmHg), median (IQR)	140 (21)	136 (27)	n.s.	141 (22)	135 (27)	n.s.	136 (26)	130 (28)	n.s.

SES: socioeconomic status, WHR: waist over hip ratio, TC-HDL-C: total cholesterol over HDL cholesterol, SBP: systolic blood pressure. ^a^Parameters for which median (IQR) is provided were log-transformed for use in linear regression models.

**Table 2:** Association between cytomegalovirus serostatus and cfPWV among the entire cohort and among men and women separately

	Beta coefficient (95% Confidence Interval)^a^			
	Model 1^b^	Model 2^c^	Model 3^d^	Model 4^e^
CMV serostatus/all (N = 136)	-0.018 (-0.019, 0.056)	-0.011 (-0.022, 0.044)	0.014 (-0.020, 0.047)	0.021 (-0.008, 0.049)^f^
**Model split by sex**				
CMV serostatus/men (N = 72)	0.046 (0.001, 0.092)*	0.048 (0.008, 0.087)*	0.055 (0.016, 0.094)**	0.059 (0.024, 0.093)^g^**
CMV serostatus/women (N = 64)	-0.016 (-0.078, 0.046)	-0.027 (-0.081, 0.027)	-0.024 (-0.079, 0.031)	-0.010 (-0.055, 0.035)^h^

^a^log10 units; ^b^Model 1 unadjusted model; ^c^Model 2 adjusted for age, socioeconomic status, and sex (entire cohort only); ^d^Model 3 additionally adjusted for smoking pack-years and WHR; ^e^Model 4 additionally adjusted for lying SBP and total TC/HDL-C; ^f^R-squared of fully adjusted model: 0.474; ^g^R-squared of fully adjusted model: 0.550; ^h^R-squared of fully adjusted model: 0.554; *P < 0.05; **P < 0.01.

**Table 3:** Association between cytomegalovirus serostatus and CD4 T_mem_ population size among the entire sub-cohort and among men and women separately

	Beta coefficient (95% Confidence Interval)^a^		
	Model 1^b^	Model 2^c^	Model 3^d^
CMV serostatus/all (N = 123)	0.048 (0.004, 0.092)*	0.056 (0.013, 0.100)*	0.053 (-0.009, 0.097)*
**Model split by sex**			
CMV serostatus/men (N = 66)	0.079 (0.027, 0.132)**	0.078 (0.024, 0.132)**	0.073 (0.18, 0.128)*
CMV serostatus/women (N = 57)	0.032 (-0.040, 0.104)	0.034 (-0.039, 0.106)	0.028 (-0.046, 0.101)

^a^log10 units; ^b^Model 1 unadjusted; ^c^Model 2 adjusted for age, socioeconomic status, and sex (entire cohort only); ^d^Model 3 additionally adjusted for smoking pack-years and WHR; *P < 0.05; **P< 0.01.

**Table 4:** Association between CD4 T_mem_ and cfPWV among the entire sub-cohort and among CMV- and CMV+ participants separately

	Beta coefficient (95% Confidence Interval)^a^				
	Model 1^b^	Model 2^c^	Model 3^d^	Model 4^e^	Model 5^f^
CD4 T_mem_ /all (N = 123)	0.045 (-0.115, 0.205)	-0.035 (-0.128, 0.199)	0.040 (-0.106, 0.187)	0.046 (-0.100, 0.193)	0.020 (-0.104, 0.144)^g^
**Models split by CMV serostatus**					
CD4 T_mem_ /CMV- (N = 51)		-0.289 (-0.561,-0.017)*	-0.143 (-0.391, 0.105)	-0.149 (-0.396, 0.099)	-0.116 (-0.337, 0.104)^h^
CD4 T_mem_ /CMV+ (N = 72)		0.249 (-0.057, 0.441)*	0.206 (0.018, 0.394)*	0.235(0.044, 0.425)*	0.181 (0.027, 0.335)^i^*

^a^log10 units; ^b^Model 1 unadjusted; ^c^Model 2 adjusted for CMV (unless stratified by CMV); ^d^Model 3 additionally adjusted for age and socioeconomic status; ^e^Model 4 additionally adjusted for smoking pack-years and WHR; ^f^Model 5 additionally adjusted for lying SBP and TC/HDL-C; ^g^R-squared of fully adjusted model: 0.472; ^h^R-squared of fully adjusted model: 0.524; ^i^R-squared of fully adjusted model: 0.528; *P < 0.05; **P < 0.01.

**Table 5:** CMV protein-covering peptide-pools used for T-cell stimulation^a^

Pool	CMV Protein(s)	No. of Peptides
1	UL55^5^	224
2	UL83	138
3	UL86	340
4	UL122	120
5	UL123	143
6	UL153	67
7	UL32	260
8	UL28	92
9	UL48A^a^	281
10	UL48B^a^	281
11	US3	44
12	UL151& UL82	219 (82 &137)
13	UL94 & US29	197 (84 &113)
14	US24 & UL36	240 (123 &117)

^a^A panel of 19 CMV protein-spanning peptide pools was previously shown to correlate highly with the CD4 and CD8 T-cell response against 203 tested CMV proteins 35. The original panel contained UL99, UL103, and US32 in addition, which had been used previously but were left out here since responses were absent in >100 White British people. ^b^48 was divided into two pools (UL48A and UL48B), however, results were combined.

## Abbreviations

cfPWV: carotid-to-femoral pulse wave velocity
CMV: cytomegalovirus
CVD: cardiovascular disease
IQR: Interquartile range
SES: socioeconomic status
TC/HDL-C: ratio of total cholesterol over high-density lipoprotein (HDL) cholesterol
T_mem_: memory T cells
WHR: waist-to-hip ratio
