# Supplementary material for: Cytomegalovirus infection is associated with an increase in aortic stiffness in older men which may be mediated in part by CD4 memory T-cells
Source: Theranostics. 2021 Mar 31;11(12):5728–41. doi: 10.7150/thno.58356 (PMC8058738; doi:10.7150/thno.58356)
Supplement: Supplementary file 1 — Supplementary figures and tables. [file thnov11p5728s1.pdf]

Supplementary Materials

Cytomegalovirus infection is associated with an increase in aortic stiffness in older men which may be mediated in part by CD4 memory T-cells

Kirkham, F. et al.

Materials and methods

Participants and samples

GPs were instructed to search their electronic patient records with respect to the study's inclusion and exclusion criteria, compile a list of all potentially eligible participants, assign a number to each individual, and communicate the total number eligible to the PCRN.

Over several recruitment waves the PCRN provided each practice with a list of random numbers. Larger practices were instructed to reach out to 100 individuals per wave and engaged in more waves than smaller practices, which were instructed to reach out to 50 individuals per wave. For each wave of invitations, new random numbers were sent to each practice (previously used numbers were excluded). Recruitment occurred in multiple waves over a total of 2.5 years.

Practices mailed written invitations to all selected individuals. If interested, invited participants contacted the research nurse team at the Clinical Investigation and Research Unit (CIRU) of Brighton and Sussex University Hospital NHS Trust (BSUHT) to schedule an initial screening appointment, which took place in their GP's practice. At this appointment, inclusion/exclusion criteria were double-checked, written informed consent was obtained from eligible participants, a blood sample was obtained by venepuncture for testing CMV-serostatus, and participants were invited to attend the main study appointment at CIRU.

Participants from a previous study (N = 131) using the same random recruitment approach 3 years earlier, were invited as well (direct mail-out by CIRU). Respondents (N = 30) were invited directly for the main appointment after confirming eligibility via telephone. Their CMV status had previously been recorded but was not a criterion for selection.

At the main study appointment, eligibility of all participants was reviewed, a detailed medical history was obtained, vascular measurements were performed, and additional blood samples were taken for measuring blood counts, lipid values, and general blood biochemistry.

Individuals were recruited in the order in which responses were received. Vascular laboratory staff were blinded to the CMV status of participants at all times. Recruitment was continued

until N = 209 individuals had attended the main appointment. After reviewing eligibility according to the selection criteria N = 193 participants were eligible for assessment in the vascular lab (Figure S5). Of these, 104 were CMV+ and 89 were CMV-.

cfPWV

Briefly, with the patient in supine position the carotid and femoral arteries are located and the positions where carotid and femoral pressures are to be measured are marked. The distance between these points is measured and recorded in the analysis software. The sensors are positioned on the respective measurement points for data acquisition. During measurement, the time-delay between recording the pulse wave at the carotid and femoral arteries is recorded along with the changes in central and peripheral brachial blood pressure. The analysis software subsequently displays pulse wave velocity and the central (carotid) pressure waveform analysis. Measurement of cfPWV was attempted in 193 and completed in 181 individuals. In 12 individuals the procedure was aborted due to compliance issues. A total of N = 137 (76%) of cfPWV measurements passed quality control (QC) by two physicians blinded to the CMV infection status of participants. QC was based on the shape of the recordings and the consistency (wave form) of the ten consecutive waves. Extreme outliers in terms of cfPWV (> LQ- (3*IQR); < (UQ+3*IQR) were removed during analysis leaving N = 136 accepted measurements for cfPWV.

Whole blood antibody staining for T-cell phenotyping

Fresh, sodium-heparin-anticoagulated whole blood (100 µL) was incubated with monoclonal, fluorescence-labeled staining antibodies as follows. Anti-CD45-AlexaFluor (AF)700, anti-CD3-Pacific Blue (PB), anti-CD4-Brilliant Violet (BV)510, anti-CD8α-allophycocyanin (APC)-H7, anti-CCR7 BV605, anti-CD45RA-Fluorescein-iso-thio-cyanate (FITC) (all BioLegend, Cambridge, UK). Cells were incubated for 20 min at RT in the dark followed by addition of BD lysis buffer (BD) and incubation for 10 min according to the manufacturer's instructions. Samples were then centrifuged (5 min at 350*g*), resuspended in PBS and spun again (5 min at 350*g*). Finally, cells were fixed (1% paraformaldehyde in water) and washed. Pellets were resuspended in 300 µL of staining buffer (BD) prior to acquisition within 1-4 h.

CMV-peptides

Twenty-five µg per peptide per pool (PepMix^{TM}, JPT Peptide Technologies, Berlin, Germany)

were dissolved in 100 μL of DMSO (Sigma-Aldrich, Gillingham, UK) (0.25 μg/μL). For T-cell stimulation assays 2 μL of the 0.25 μg/μL CMV-peptide solutions were used for a final concentration of 1 μg/mL per peptide. All assays included a positive control that consisted of 1 μg/ml (final concentration) of Staphylococcal enterotoxin B from *Staphylococcus aureus* (SEB, Sigma) dissolved in DMSO, and a negative control consisting of 2 μL of DMSO alone (i.e., the peptide solvent). A list of all CMV proteins used in the study is provided in **Table 5**.

CMV-reactivity of T-cells

CMV-specific T-cells from CMV+ individuals were activated by incubating PBMC for 16 h overnight (37 °C, humidified 5% CO_2 atmosphere) with peptide pools, positive and negative controls. Per tube $1x10^6$ PBMCs were incubated with 2 μL of the corresponding CMV peptide pool, 1 μL of SEB solution (positive control, final concentration 1 μg/mL) or 2 μL of DMSO (negative control) in complete RPMI media (final incubation volume 250 μL). After 2 h in a standard incubator (37°C, humidified 5% CO2 atmosphere) each tube received 250 μL of complete RPMI containing 10 μg/mL of Brefeldin A (BFA), resulting in a final volume of 500 μL for the remaining incubation time (14 h). Thereafter each tube was incubated for 10 min at 37 °C (water bath) with 100 μL of 20 mM EDTA (Sigma). Subsequently, samples were washed with 3 mL of wash buffer (PBS with 0.5% w/v BSA and 0.1% w/v sodium azide, (Sigma) and centrifuged at 400*g* for 8 min at RT ('wash step'). Samples were then surface-stained (30 min at 4 °C in the dark) with fluorochrome conjugated monoclonal antibodies including anti-CD3 Pacific Blue, anti-CD4 BV510, anti-CD8a PE-Cy7, anti-CCR7 BV605, and anti-CD45RA PerCP/Cy5.5 (all Biolegend), followed by a wash step. Following the surface stain 1ml of 1x BD 'FACS Lysing Solution' (BD) was added to each tube and tubes incubated for 10 min in the dark. Another wash step followed and subsequently, BD 'Permeabilization solution 2' was added to each tube and samples were incubated for 10 min in the dark. After a further wash step, cells were stained intracellularly with anti-IL-2 APC, anti-TNFα AF 700 and anti-IFN-γ FITC (all Biolegend) for 30 min at 4 °C in the dark followed by a wash step. Then, 1 mL of 0.5% paraformaldehyde (in water) was added to each tube and incubated in the dark for 5 min. After a final wash step cells were stored at 4 °C in the dark until acquisition.

Data acquisition and analysis

Samples were acquired on an LSR II flow-cytometer (BD). FlowJo v9.x software (Tree Star Inc., Ashland, OR) was used for data analysis. The gating strategies for phenotype staining of

whole blood samples and intracellular cytokine staining for PBMC samples are described in Figures S3 and S6, respectively.

Quality control for flow-cytometry

Comparable day-to-day performance of the LSR II flow-cytometer was ascertained by running CS&T calibration beads (BD) on a daily basis. In addition, 8-peak Rainbow beads were used prior to every run in order to adjust PMTs in such a way that with respect to each detector, every peak was always in the same channel.

Statistical analysis

Linear regression models were adjusted for covariates as follows.

(1) Linear regression models testing an association of CMV infection status (CMV+ or CMV-) with cfPWV. First, models were unadjusted. They were then adjusted for sex (total sample only), age, and SES. Next, they were adjusted smoking pack-years and WHR. Finally, models were adjusted for SBP and TC/HDL-C.

(2) Linear regression models testing an association of CMV infection status with T_{mem}. Initial models were unadjusted. Subsequent models were adjusted for age, SES, and sex (unless stratified by sex). They were then adjusted for smoking pack-years and WHR. Models were not adjusted for SBP and TC/HDL-C as no consistent effect of these parameters on T_{mem} is known.

(3) Linear regression models testing an association of T_{mem} with cfPWV. Initial models were unadjusted. Subsequent models were adjusted for CMV (unless stratified by CMV), and then age (unless stratified by age) and SES. Finally, models were adjusted for WHR and smoking pack-years. Finally, models were adjusted for SBP and TC/HDL-C. To avoid collinearity, these models were not adjusted for sex, because the effect of sex was already accounted for by the proportions of T_{mem} (which were higher in men than in women).

Data trends in scatter plots were visualised using the SPSS ‘fit line’ command (linear model). Bivariate correlations were tested using the ‘correlate’ command in SPSS, the Pearson Correlation Coefficient (R) is reported.

Supplementary Figures

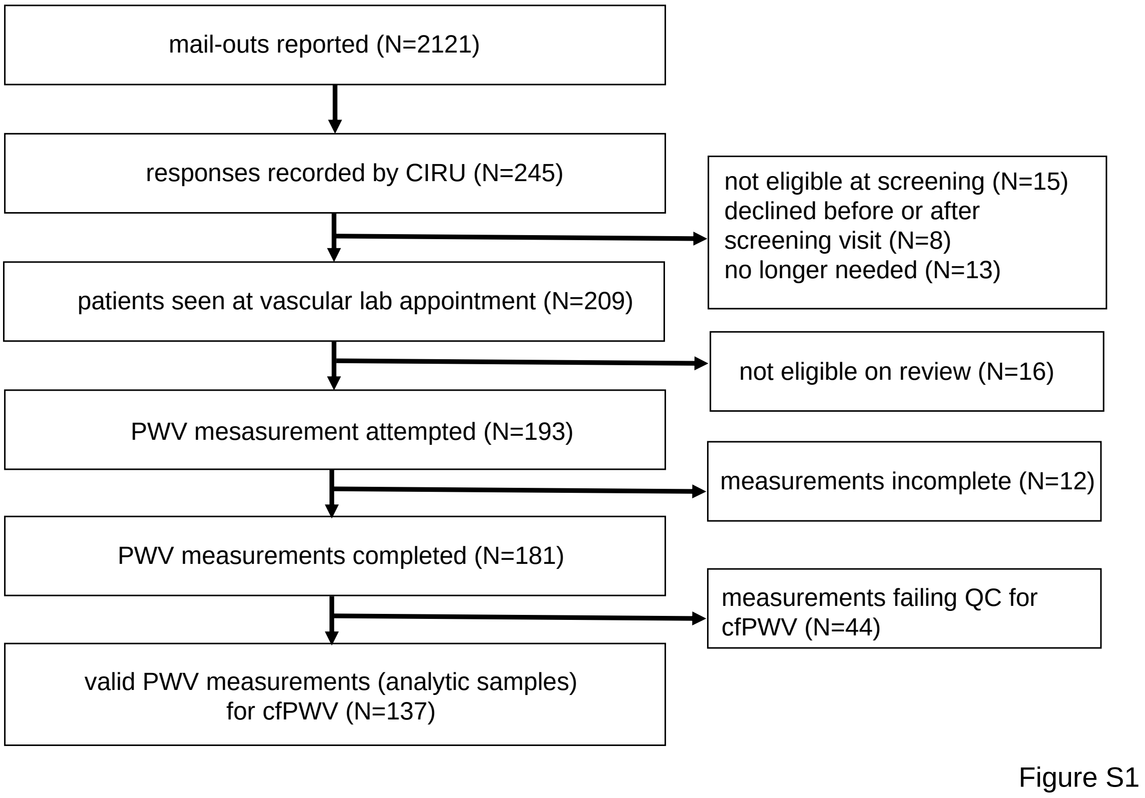

Figure S1. Flowchart describing the recruitment of individuals for measuring PWV and other relevant parameters.

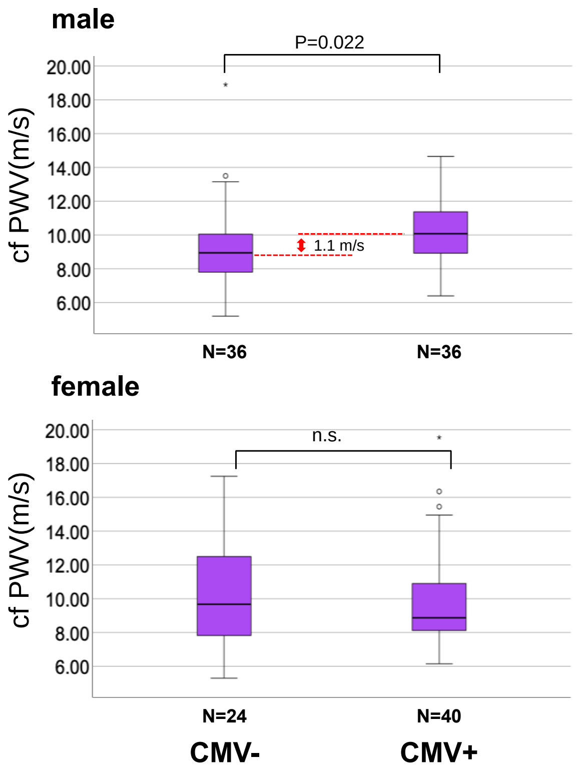

Figure S2

Figure S2. cfPWV is higher in CMV+ compared with CMV- older men. Diagrams show the distribution of cfPWV in men and women stratified by CMV infection status. Box plots show median and interquartile range, outlier limits (whiskers, LQ-1.5*IQR, UQ+1.5*IQR), outliers (o) and extreme values (*). Significant differences are indicated by P-values.

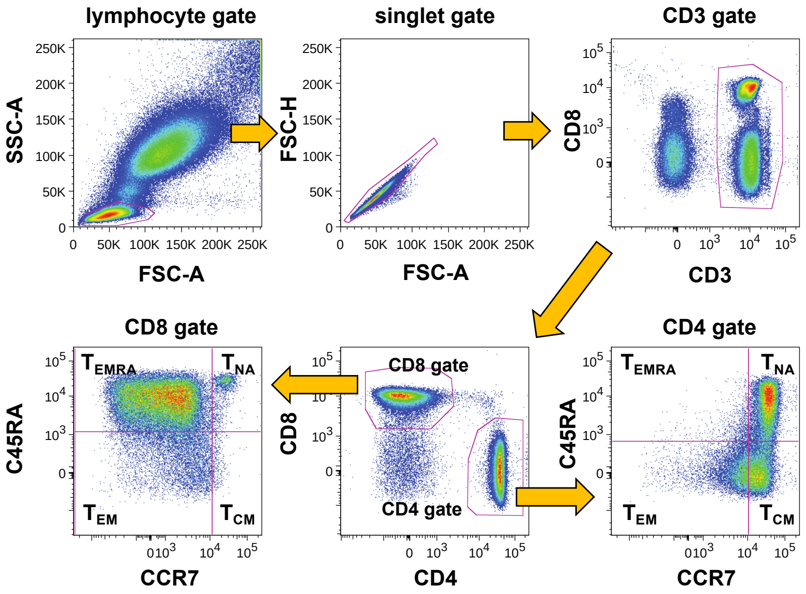

Figure S3

Figure S3. Gating strategy for whole blood surface phenotyping. Lymphocytes were gated in an SSC/FSC plot. Singlets were gated in an FSC-A/FSC-H plot, doublets were excluded. From the singlet gate a CD3 T-cell gate was created in a CD3/CD8 fluorescence dot-plot (top right). A CD4 versus CD8 fluorescence dot plot allowed to visualize T-cell subsets based on CD4 and CD8 expression (middle). For the analysis of CCR7 and CD45RA expression CD4 T-cells (bottom left) and CD8 T-cells (bottom right) were gated separately. The canonical naïve (CCR7+CD45RA+, T_{NA}), central memory (CCR7+CD45RA-, T_{CM}), effector memory (CCR7-CD45RA-, T_{EM}), and revertant memory T-cell subsets (CCR7-CD45RA+, T_{EMRA}) were identified as shown. The same gating sequence and strategy was applied to all samples. The expression of CD28 was used to aid the discrimination between the subsets in that the CCR7+/CD45RA+ subset is $CD28^{bright}$ (not shown).

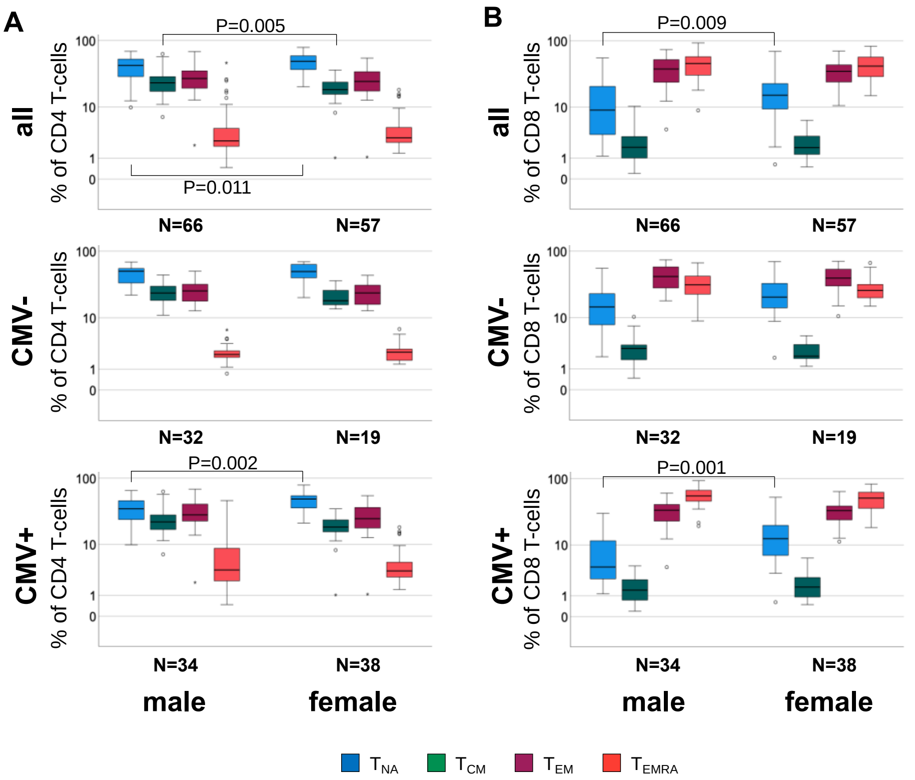

Figure S4

Figure S4. Differential distribution of canonical CD4 and CD8 memory T-cell subsets in men and women stratified by CMV status. A. Box plots show the distribution of CD4 T_{NA}, T_{CM}, T_{EM}, and T_{EMRA} subsets in men and women among the entire cohort (top), CMV- individuals (middle) and CMV+ individuals (bottom). B. Box plots show the distribution of the corresponding CD8 T cell subsets in each group. Box plots show median and interquartile range, outlier limits (whiskers, LQ-1.5*IQR, UQ + 1.5*IQR), outliers (o) and extreme values (*). Significant differences are indicated by P-values.

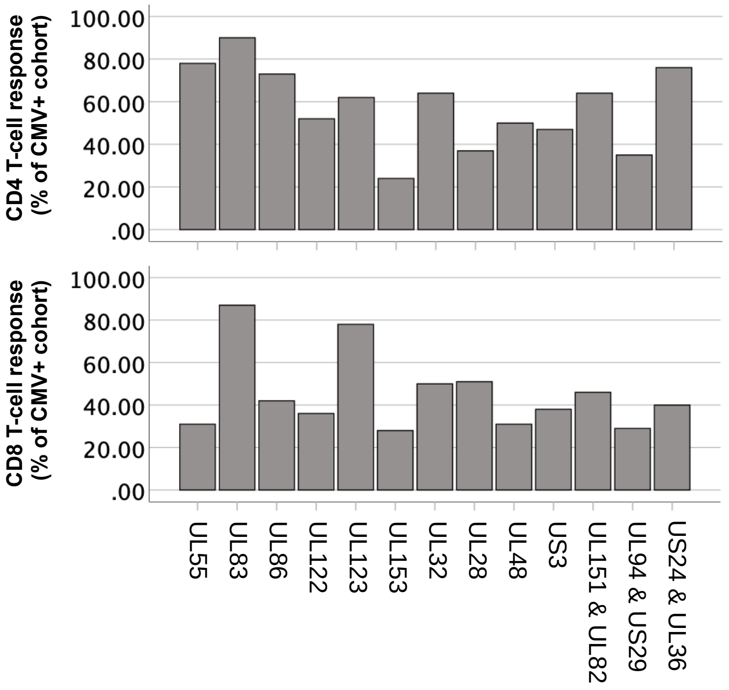

Figure S5

Figure S5: Bar charts showing the distribution of CD4 (top) and CD8 (bottom) T-cell responsiveness to 16 different CMV proteins arranged in 14 stimulation pools. Each column represents the number of positive responses among CMV+ people to one stimulation pool. Positive responses were identified by intracellular cytokine staining following stimulation of fresh PBMC with protein-spanning peptide pools.

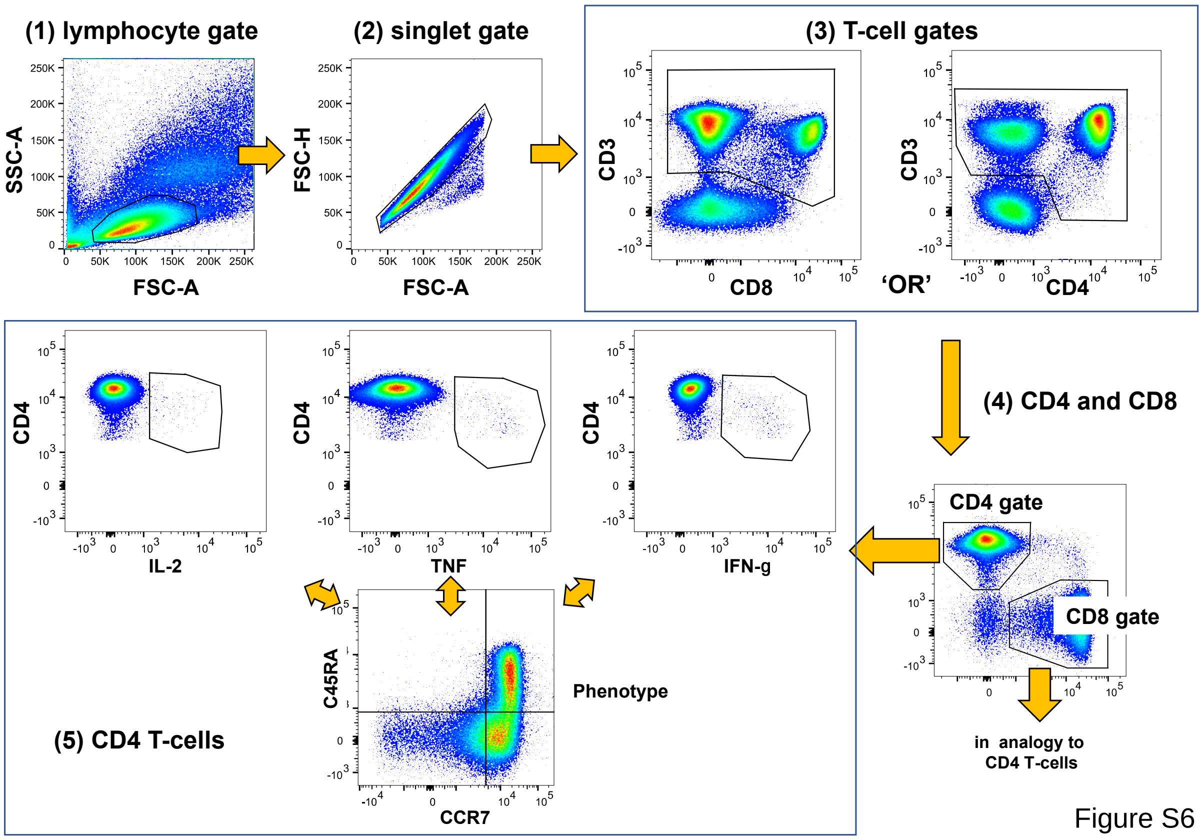

Figure S6. Gating strategy for ICS on antigen-stimulated, fresh PBMC. (1) Lymphocytes were gated in an SSC/FSC plot. (2) Singlets were gated in an FSC-A/FSC-H plot, and doublets were excluded. (3) From the singlet gate, two CD3 T-cell gates were created, one in a CD3/CD4 fluorescence dot-plot (top right) and one in a CD3/CD8 fluorescence dot-plot. Each gate allowed for inclusion of sometimes strongly TCR downregulated T-cells. (4) The two CD3 gates were then combined in a Boolean gate (OR) for gating CD4 and CD8 T-cells in a CD4 versus CD8 fluorescence dot plot, which allowed to visualize T-cell subsets based on CD4 and CD8 expression. (5) For the analysis of activated T-cells, CD4 and CD8 T-cells were plotted versus the activation markers (IL-2, TNF, and IFN-γ). These gates were overlaid with the canonical memory T-cell subsets defined by CCR7 and CD45RA expression. The same gating sequence and strategy was applied to all samples.

Supplementary Tables

Table S1. Association between cytomegalovirus serostatus and cfPWV among men and women separately, fully adjusted model

	**Beta coefficient (95% Confidence Interval)[a]**	**P value**
**Men[b]**		
**CMV serostatus**	0.059 (0.024, 0.093)	0.001
**Age**	0.005 (0.003, 0.008)	0.000
**SES**	-0.087 (-0.163, -0.012)	0.024
**WHR**	0.142 (0.017, 0.267)	0.027
**Smoking pack-years**	0.000 (-0.006, -0.006)	0.961
**TC/HDL-C**	-0.006 (-0.026, 0.014)	0.562
**SBP lying**	0.732 (0.422, 1.041)	0.001
**Women[c]**		
**CMV serostatus**	-0.010 (-0.055, 0.035)	0.661
**Age**	0.007 (0.003, 0.011)	0.001
**SES**	0.064 (-0.037, 0.165)	0.212
**WHR**	-0.219 (-0.556, 0.118)	0.198
**Smoking pack-years**	0.007 (0.000, 0.015)	0.060
**TC/HDL-C**	-0.004 (-0.024, 0.032)	0.776
**SBP lying**	1.000 (0.641, 1.359)	0.000

SES: socioeconomic standing, WHR: waist over hip ratio, TC-HDL-C: total cholesterol over HDL cholesterol, SBP:systolic blood pressure

[a]log10 units

[b]R-squared of fully adjusted model: 0.550

[c]R-squared of fully adjusted model: 0.554

Table S2. Additional information on smoking and blood pressure (entire cohort)

	All			Male			Female		
	**CMV-** (N = 60)	**CMV+** (N = 76)	**P**	**CMV-** (N = 36)	**CMV+** (N = 36)	**P**	**CMV-** (N = 24)	**CMV+** (N = 40)	**P**
**DBP lying (mmHg) median (IQR)**	76.8 (11.1)	74.5 (14.5)	n.s.	79.8 (9.1)	76.0 (19.3)	n.s.	73.8 (9.6)	73.8 (14.1)	n.s.
**PP lying (mmHg) median (IQR)**	58.0 (18.9)	57.2 (18.1)	n.s.	57.5 (16.0)	58.0 (15.4)	n.s.	61.8 (19.5)	55.0 (23.1)	n.s.
**Ever smoker N (% in column)**	32 (53.3)	43 (57.6)	n.s.	19 (52.8)	23 (63.9)	n.s.	13 (54.2)	20 (50.0)	n.s.
**Current smoker N (% in column)**	6 (10.0)	5 (6.5)	n.s.	5 (13.8)	3 (8.3)	n.s.	1 (4.1)	2 (5.0)	n.s.
**Time since smoking cessation in ex-smokers, mean ± STD**	31.7 ± 14.2	34.1 ± 13.2	n.s.	29.6 ± 14.5	31.0 ± 14.2	n.s.	34.0 ± 14.0	37.5 ± 11.5	n.s.

DBP = diastolic blood pressure, PP = pulse pressure

Table S3. Common medications[a,b] (entire cohort)

	All			Male			Female		
	**CMV- (N = 60)**	**CMV+ (N = 76)**	**P**	**CMV- (N = 36)**	**CMV+ (N = 36)**	**P**	**CMV- (N = 24)**	**CMV+ (N = 40)**	**P**
**Cholesterol lowering drugs (all)**	17 (28.3)	14 (18.4)	n.s.	14 (38.9)	8 (22.2)	n.s.	3 (12.5)	6 (15.0)	n.s.
**Statin**	16 (26.7)	14 (18.4)	n.s.	13 (36.1)	8 (22.2)	n.s.	3 (12.5)	6 (15.0)	n.s.
**Oral anti-hyperglycemic**	3 (5.0)	0 (0.0)	n.s.	1 (2.8)	0 (0.0)	n.s.	2 (8.3)	0 (0.0)	n.s.
**Blood pressure lowering drugs (any)**	18 (30)	22 (28.9)	n.s.	11 (30.6)	9 (25.0)	n.s.	7 (29.2)	13 (32.5)	n.s.
**Blood pressure lowering drugs (number) mean± STD**	0.43 ± 0.77	0.49 ± 0.92	n.s.	0.44± 0.77	0.47± 0.97	n.s.	0.42± 0.77	0.50± 0.87	n.s.
**Diuretic**	3 (5.0)	8 (10.5)	n.s.	1 (2.8)	3 (8.3)	n.s.	2 (8.3)	5 (12.5)	n.s.
**Beta-blocker**	8 (13.3)	5 (6.6)	n.s.	5 (13.9)	2 (5.6)	n.s.	3 (12.5)	3 (7.5)	n.s.
**Ca-antagonist**	6 (10.0)	5 (6.6)	n.s.	4 (11.1)	3 (8.3)	n.s.	2 (8.3)	2 (5.0)	n.s.
**ACE inhibitor/AT-II receptor antagonist**	12 (20)	16 (21.1)	n.s.	8 (22.2)	7 (19.4)	n.s.	4 (16.7)	9 (22.5)	n.s.
**Centrally acting anti-hypertensive**	0 (0.0)	1 (1.3)	n.s.	0 (0.0)	1 (2.8)	n.s.	0 (0.0)	0 (0.0)	n.s.
**Nitroglycerine**	0 (0.0)	0 (0.0)	n.s.	0 (0.0)	0 (0.0)	n.s.	0 (0)	0 (0.0)	n.s.
**Platelet aggregation inhibitor**	6 (10.0)	9 (11.8)	n.s.	5 (13.9)	7 (19.4)	n.s.	1 (4.2)	2 (5.0)	n.s.

[a]Medications beyond the listed categories included commonly prescribed drugs for the management of chronic pain, hypothyroidism, osteoporosis, COPD, asthma, allergies, constipation, skin conditions, and gastritis/ulcers.
[b]Unless indicated otherwise, values are reported as N (% of group in column)

Table S4. Association between cytomegalovirus serostatus and cfPWV among the entire cohort and among men and women separately

	Beta coefficient (95% Confidence Interval)[a]	
	**Model 4A[b]**	**Model 4B[c]**
**CMV serostatus/all (N = 136)**	0.021 (-0.008, 0.049)	0.022 (-0.006, 0.050)
**Models split by gender:**		
**CMV serostatus/men (N = 72)**	0.059 (0.024, 0.093)**	0.059 (0.024, 0.094)**
**CMV serostatus/women (N = 64)**	-0.011 (-0.056, 0.035)	-0.011 (-0.056, 0.033)

[a]log10 units
[b]Model 4A corresponds to Model 4 in Table 2, additionally adjusted for the number of anti-hypertensive medications taken.
[c]Model 4B corresponds to Model 4 in Table 2, additionally adjusted for presence/absence of antihypertensive medication (yes/no)
*P < 0.05; **P < 0.01

Table S5. Characteristics used in linear regression models (sub-cohort with T-cell phenotyping data)[a]

	All			Male			Female		
	**CMV- (N = 51)**	**CMV+ (N = 72)**	**P**	**CMV- (N = 32)**	**CMV+ (N = 34)**	**P**	**CMV- (N = 19)**	**CMV+ (N = 38)**	**P**
**PWV (m/s) median (IQR)**	9.5 (3.4)	9.4 (3.0)	n.s.	8.9 (2.9)	10.1 (2.5)	0.029	9.8 (4.3)	8.9 (3.3)	n.s.
**Age years mean ± STD**	69.1 ± 7.4	70.4 ± 7.2	n.s.	69.7 ± 7.8	71.0 ± 8.7	n.s.	68.1 ± 6.8	69.8 ± 5.6	n.s.
**SES median (IQR)**	4.3 (3.9)	6.0 (4.8)	n.s.	4.2 (3.9)	6.0(5.2)	n.s.	5.0 (3.0)	4.7 (3.1)	n.s.
**Smoking (pack-years) median (IQR)**	0.5 (13.0)	0.1 (6.8)	n.s.	1.3 (12.5)	1.1 (23.1)	n.s.	0.5 (15.0)	0.0 (3.2)	n.s.
**WHR mean ± STD**	1.0 ± 0.2	0.9 ± 0.1	n.s.	1.0 ± 0.20	1.0 ± 0.1	n.s.	0.9 ± 0.0	0.9± 0.1	n.s.
**TC/HDL-C mean ± STD**	3.4 ± 0.9	3.2 ± 0.8	n.s.	3.5 ± 0.9	3.3 ± 0.8	n.s.	3.1 ± 1.0	3.0 ± 0.8	n.s.
**SBP lying (mmHg) median (IQR)**	142 (18)	135 (26)	n.s	141 (16)	137 (24)	n.s	142 (25)	131 (28)	n.s

SES: socioeconomic standing, WHR: waist over hip ratio, TC-HDL-C: total cholesterol over HDL cholesterol, SBP: systolic blood pressure
[a]Parameters for which median (IQR) is provided were log-transformed for use in linear regression models

Table S6. Additional information on smoking and blood pressure (sub-cohort with T-cell phenotyping data)

	All			Male			Female		
	**CMV- (N=51)**	**CMV+ (N=72)**	**P**	**CMV- (N=32)**	**CMV+ (N=34)**	**P**	**CMV- (N=19)**	**CMV+ (N=38)**	**P**
**DBP lying (mmHg) median (IQR)**	77.5 (10.0)	74.8 (14.8)	n.s	80.3 (9.0)	77.0 (20.1)	n.s.	74.5 (10.0)	73.8 (14.1)	n.s.
**PP lying (mmHg) median (IQR)**	58.5 (18.5)	57.3 (18.1)	n.s.	57.5 (12.9)	58.8 (14.4)	n.s.	68.5 (21.5)	54.0 (23.1)	n.s.
**Ever smoker N (% in column)**	28 (55.0)	39 (54.1)	n.s.	18 (56.2)	21 (61.8)	n.s.	10 (52.3)	18 (47.3)	n.s.
**Current smoker N (% in column)**	5 (9.8)	4 (5.6)	n.s.	4 (12.5)	2 (5.9)	n.s.	1 (5.3)	2 (5.3)	n.s.
**Time since smoking cessation in ex-smokers, mean ± STD**	30.8 ± 14.9	33.9 ± 13.8	n.s.	29.6 ± 14.5	30.8 ± 14.5	n.s.	32.7 ± 16.1	37.7 ± 12.2	n.s.

DBP = diastolic blood pressure, PP = pulse pressure

Table S7. Common medications[a,b] (sub-cohort with T-cell phenotyping data)

	All			Male			Female		
	**CMV- (N = 51)**	**CMV+ (N = 72)**	**P**	**CMV- (N = 32)**	**CMV+ (N = 34)**	**P**	**CMV- (N = 19)**	**CMV+ (N = 38)**	**P**
**Cholesterol lowering drugs (all)**	15 (29.4)	14 (19.4)	n.s.	12 (37.5)	8 (23.5)	n.s.	3 (15.8)	6 (15.8)	n.s.
**Statin**	14 (27.5)	14 (19.4)	n.s.	11 (34.4)	8 (23.5)	n.s.	3 (15.8)	6 (15.8)	n.s.
**Oral anti-hyperglycemic**	3 (5.9)	0 (0.0)	n.s.	1 (3.1)	0 (0.0)	n.s.	2 (10.5)	0 (0.0)	n.s.
**Blood pressure lowering drugs (any)**	16 (31.4)	21 (29.2)	n.s.	10 (31.3)	9 (26.5)	n.s.	6 (31.6)	12 (31.6)	n.s.
**BP medications (No) mean± STD**	0.41 ± 0.70	0.49 ± 0.91	n.s.	0.44 ± 0.75	0.50 ± 0.99	n.s.	0.37 ± 0.59	0.47 ± 0.86	n.s.
**Diuretic**	2 (3.9)	7 (9.7)	n.s.	1 (3.1)	3 (8.8)	n.s.	1 (5.3)	4 (10.5)	n.s.
**Beta-blocker**	6 (11.8)	5 (6.9)	n.s.	4 (12.5)	2 (5.9)	n.s.	2 (10.5)	3 (7.9)	n.s.
**Ca-antagonist**	6 (11.8)	5 (6.9)	n.s.	4 (12.5)	3 (8.8)	n.s.	2 (10.5)	2 (5.3)	n.s.
**ACE inhibitor/AT-II receptor antagonist**	10 (19.6)	15 (20.8)	n.s.	7 (22.2)	7 (21.9)	n.s.	3 (15.8)	8 (21.1)	n.s.
**Centrally acting anti-hypertensive**	0 (0.0)	1 (1.4)	n.s.	0 (0.0)	1 (2.9)	n.s.	0 (0.0)	0 (0.0)	n.s.
**Nitroglycerine**	0 (0.0)	0 (0.0)	n.s.	0 (0.0)	0 (0.0)	n.s.	0 (0.0)	0 (0.0)	n.s.
**Platelet aggregation inhibitor**	4 (7.8)	9 (12.5)	n.s.	3 (9.4)	7 (20.6)	n.s.	1 (5.3)	2 (5.3)	n.s.

[a]Medications beyond the listed categories included commonly prescribed drugs for the management of chronic pain, hypothyroidism, osteoporosis, COPD, asthma, allergies, constipation, skin conditions, and gastritis/ulcers.
[b]unless indicated otherwise, values are reported as N (% of group in column)

Table S8. Association between cytomegalovirus seropositivity and CD8 T_{mem} population size among the entire sub-cohort with phenotyping data and among men and women separately

**Sample**	**Beta coefficient (95% Confidence Interval)[a]**		
	**Model 1[b]**	**Model 2[c]**	**Model 3[d]**
**CMV serostatus/all (N = 123)**	0.058 (0.029, 0.086)***	0.063 (0.034, 0.092)***	0.064 (0.034, 0.093)***
**Models split by gender:**			
**CMV serostatus/men (N = 66)**	0.059 (0.025, 0.092)**	0.059 (0.025, 0.093)**	0.060 (0.025, 0.094)**
**CMV serostatus/women (N = 57)**	0.070 (0.020, 0.119)**	0.072 (0.022, 0.123)**	0.073 (0.021, 0.125)**

[a]log10 units
[b]Model 1 unadjusted.
[c]Model 2 adjusted for age, socioeconomic status, and sex (total sample only).
[d]Model 3 additionally adjusted for smoking pack-years and waist-to-hip ratio.
*P < 0.05, **P < 0.01, ***P < 0.001
